# *Corynebacterium pseudotuberculosis* cp09 mutant and cp40 recombinant protein partially protect mice against caseous lymphadenitis

**DOI:** 10.1186/s12917-014-0304-6

**Published:** 2014-12-20

**Authors:** Judson W Silva, Daniela Droppa-Almeida, Sibele Borsuk, Vasco Azevedo, Ricardo W Portela, Anderson Miyoshi, Flávia S Rocha, Fernanda A Dorella, Wanessa L Vivas, Francine F Padilha, Maria L Hernández-Macedo, Isabel B Lima-Verde

**Affiliations:** Technology and Research Institute, Tiradentes University, Av. Murilo Dantas, 300, Aracaju, Sergipe 49032-490 Brazil; Biotechnology Unit/Center for Technology Development, Federal University of Pelotas, Capão do Leão, Rio Grande do Sul 96010-900 Brazil; Biological Sciences Institute, General Biology Department, Federal University of Minas Gerais, Av. Antônio Carlos, 6627, Pampulha, Minas Gerais Belo Horizonte, Brazil; Health Sciences Institute, Federal University of Bahia, Avenida Reitor Miguel Calmon s/n, Salvador, BA 40110-100 Brazil

**Keywords:** Caseous lymphadenitis, *Corynebacterium pseudotuberculosis*, Recombinant vaccines, Live attenuated vaccines

## Abstract

**Background:**

Caseous lymphadenitis (CLA) is an infectious disease that affects small ruminants and is caused by *Corynebacterium pseudotuberculosis*. This disease is responsible for high economic losses due to condemnation and trim of infected carcasses, decreased leather and wool yield, loss of sales of breeding stock and deaths from internal involvement. Treatment is costly and ineffective; the most cost-effective strategy is timely immunisation. Various vaccine strategies have been tested, and recombinant vaccines are a promising alternative. Thus, in this study, different vaccine formulations using a recombinant protein (rCP40) and the CP09 live recombinant strain were evaluated. Five groups of 10 mice each were immunised with saline (G1), rCP40 (G2), CP09 (G3), a combination of CP09 and rCP40 (G4) and a heterologous prime-boost strategy (G5). Mice received two immunisations within 15 days. On day 30 after primary immunisation, all groups were challenged with a *C. pseudotuberculosis* virulent strain. Mice were monitored and mortality was recorded for 30 days after challenge.

**Results:**

The G2, G4 and G5 groups showed high levels of IgG1 and IgG2a; G2 presented significant IgG2a production after virulent challenge in the absence of IgG1 and IgG3 induction. Thirty days after challenge, the mice survival rates were 20 (G1), 90 (G2), 50 (G3), 70 (G4) and 60% (G5).

**Conclusions:**

rCP40 is a promising target in the development of vaccines against caseous lymphadenitis.

## Background

Caseous lymphadenitis (CLA) is an infectious, chronic and subclinical disease of sheep and goats with a worldwide occurrence [1,2]. CLA is characterised by the formation of subcutaneous abscesses in lymph nodes or as a subclinical infection in internal organs, such as the lungs, liver and kidneys [3]. The etiologic agent of CLA is *Corynebacterium pseudotuberculosis*, a gram-positive, non-spore-forming, aerobic and facultative intracellular parasite of macrophages [3]. CLA is responsible for significant economic losses in the sheep and goat industries due to its high incidence, causing condemnation of carcasses, devaluation of leather due to scars left by abscesses, decreased production of meat or milk, and occasional death of affected animals [4,5].

CLA commercial vaccines are based on attenuated strains of *C. pseudotuberculosis* and do not provide satisfactory protection for goats. Additionally, annual boosters are needed, which makes prophylaxis more costly [6]. Thus, the search for an ideal CLA vaccine has been prioritised, with the aim of obtaining long-lasting protection with few side effects. Additionally, the vaccine should allow the differentiation between vaccinated and infected animals [1].

Pursuing this purpose, different vaccine strategies have been tested, and live attenuated vaccines and recombinant proteins are promising options. Through a process of random mutagenesis, it was identified 34 live recombinant strains of *C. pseudotuberculosis* [7], one of which (CZ171049, now called CP09) presented promising results in pilot trials, where inoculated mice presented no clinical signs of caseous lymphadenitis and produced a significant amount of specific IgG.

The serine protease CP40, encoded by the *cp40* gene of *C. pseudotuberculosis*, was tested in infected animals and demonstrated immunogenic properties, showing a strong humoral response on immunoblots and a 82% reduction in the proportion of infected sheep and a 98% reduction in lung lesions [8]; these protection levels were similar to those found with other immunogens, as inactivated bacteria and genetically detoxified phospholipase D [9,10].

Considering the need for new immunogens for caseous lymphadenitis prophylaxis and the promising results already found with the mutant CP09 strain and the recombinant CP40 protein (rCP40), the aim of this work is to perform a comparative evaluation of four vaccine formulations containing these immunogens alone or in combination, and determine their ability to induce immunoprotection against caseous lymphadenitis in a murine model.

## Methods

### *C. pseudotuberculosis* strains and culture conditions

The previously obtained *C. pseudotuberculosis* CP09 mutant strain [7], the T1 pathogenic wild-type parental strain [11], and the caprine-pathogenic strain MIC-6 from the Laboratory of Genetics and Control of Microorganisms (Belo Horizonte, Brazil) bacterial collection were employed in this work. Strains were aerobically grown in “Brain Heart Infusion” broth (BHI, Oxoid) at 37°C. Kanamycin (kanamycin sulphate 25 μg/mL; Sigma-Aldrich, USA) was added to the mutant growth media. T1 and MIC-6 strains were isolated from caseous lesions of goats and have previously been employed in vaccination trials using goats and mice [11,12].

### Bacterial interaction assays with murine J774 cells

Pre-macrophagic J774 cells from murine lymphomas were cultivated in Dulbecco’s modified Eagle’s essential medium (DMEM, Sigma-Aldrich, USA) supplemented with 5% foetal bovine serum, 50 μg/mL gentamicin and 2.5 μg/mL fungizone at 37°C in a 5% CO_2_ atmosphere. The CP09 mutant strain and T1 wild-type strain were grown for 48 hours at 37°C and washed three times with PBS, resuspended in DMEM to a concentration of 10^6^ CFU/mL, and used to infect J774 cells (multiplicity of infection [MOI]: 10 bacteria: 1 cell) grown to approximately 95% confluence in 24-well tissue culture plates.

For determination of intracellular viable bacteria, after 1, 3 and 6 hours of incubation, infected J774 monolayers were washed six times with PBS and treated with 150 μg/mL gentamicin sulphate (Sigma-Aldrich, USA) diluted in DMEM for 1 h. The number of intracellular bacteria was determined by viable counts after lysis of monolayers with 0.5 mL of 0.1% TritonX-100 (Sigma-Aldrich, USA) in PBS.

### DNA extraction and PCR reaction

*C. pseudotuberculosis* genomic DNA was obtained from the T1 strain as previously described [13]. Briefly, 5.0 mL of the bacteria cultured in BHI broth was centrifuged and resuspended in 50 mM EDTA with 10 mg/mL lysozyme, and the Wizard® Genomic DNA Purification Kit (Promega, EUA) was used following the manufacturer’s instructions. After DNA extraction, the material was resolved by electrophoresis on a 1.0% agarose gel and quantified. Eluted DNA was stored at −20°C pending PCR amplification.

PCR was performed using the Go Taq® Green Master Mix Kit (Promega, EUA), with the objective to amplify the *cp40* gene (accession number NC_014329.1NCBI). The primers used in the PCR assay were CP40F (5´ CGCGGATCCATGCATAATTCTCCTCGATCAG 3’) and CP40R (5’ CGGGAATTCTTATCTAG AACCAGTTGGCTTTC 3’), which contain *Bam*HI and *Eco*RI restriction sites, respectively. Each PCR reaction had a final volume of 50 μL, with 25 μL from the kit, 1 μL of each primer (100 μM), 22 μL of ultra-purified water and 1 μL of genomic DNA from *C. pseudotuberculosis* strain T1. The amplification reaction consisted of an initial denaturation step at 94°C for 2 min, 35 cycles of denaturation for 1 min at 94°C, primer hybridisation for 1 min at 55°C and extension at 72°C for 1.5 min, followed by a final extension at 72°C for 10 min. The reaction was conducted in a thermocycler (Mastercycler Gradient, Eppendorf, Germany), and the final product was analysed by electrophoresis on a 1% agarose gel with GelRed™ (Biotium, USA) staining.

### Cloning and recombinant protein production and purification

Cloning, recombinant protein production and protein purification were performed as previously described [13]. The pAE vector [14] and the amplified *cp40* gene were digested using *BamHI* and *EcoRI* restriction enzymes (Fermentas, USA). After that, the cp40 gene was inserted into the pAE vector using the T4 DNA ligase enzyme (Fermentas, USA), and electrocompetent TOP 10 *E. coli* cells were transformed by electroporation. The transformed bacteria were cultivated in Luria-Bertani (LB) broth with 100 μg/mL ampicillin for 16 h, and the recombinant plasmid was purified using a MiniPrep Kit (Qiagen, USA). The pAE/CP40 recombinant plasmid was transformed by thermal shock in *E. coli* BL21, and the recombinant bacteria were cultivated in LB broth containing 1 mM IPTG for 3 h at 37°C in an orbital shaker.

For recombinant protein purification, the bacteria resulting from a large-scale culture (500 mL) were pelleted by centrifugation and resuspended in a washing buffer (200 mM NaH_2_PO_4_; 500 mM NaCl, 5 mM Imidazole; 8 M Urea pH 8.0) with 100 mg/mL lysozyme, sonicated five times for fifteen seconds (20 KHz) and maintained under agitation at 4°C for 16 h. The recombinant protein was purified using the HisTrap^TM^ HP (GE Healthcare, USA) affinity chromatography column followed by dialysis in a cellulose tube-based system (Sigma, USA).

### Animal model

Six- to eight-week-old BALB/c mice, a strain which is susceptible to *C. pseudotuberculosis* infection [1], were employed in this assay. For immunity tests, fifty mice were kept in a vivarium under suitable conditions of temperature and humidity with a 12 h light/dark cycle; food and water were provided *ad libitum.* The study was approved by the Ethics Committee for Animal Experimentation of the Tiradentes University (protocol number 010413).

### Immunisation protocol, challenge and determination of the protection level

The rCP40 recombinant protein and CP09 live recombinant strain were used in vaccine formulations. Animals were divided into five groups (n = 10 each) and immunised intraperitoneally (i.p.) and/or subcutaneously (s.c.) on days 0 and 15. The administration routes followed what was previously described for recombinant proteins and mutant strains immunisation protocols [7,8,10]. The negative control group (G1) was inoculated i.p. with 100 μL of saline solution (0.9% NaCl). The G2 group was inoculated i.p. with 200 μL of a solution containing rCP40 (50 μg) and saponin (7.5 μg) in sterile saline solution. G3 was inoculated s.c. with 100 μL of the CP09 recombinant live strain (10^6^ CFU). G4 was inoculated i.p. with 200 μL of a solution containing rCP40 (50 μg) and saponin (7.5 μg) and s.c. with 100 μL of the recombinant live CP09 strain (10^6^ CFU). Group G5 used a heterologous prime-boost strategy and was first inoculated i.p. with 200 μL of rCP40 (50 μg) and saponin (7.5 μg) in solution followed by an s.c. booster dose of 100 μL of the live recombinant strain CP09 (10^6^ CFU) 15 days after the first vaccination.

The previously immunised animals were challenged with 10^4^ CFU/mL (i.p.) of the MIC-6 virulent strain thirty days after the last immunisation. The protection conferred was assessed according to the survival rate of the animals immunised with different vaccine formulations. The animals were observed daily, and mortality was recorded over four weeks following the challenge.

### Blood collection and ELISA for specific IgG determination

Animals were bled at days 0, 15, 30, 45 and 60 post-immunisation to determine the antibody levels. Blood was collected from the retro-orbital sinus with Pasteur pipettes. All blood collections were performed under anaesthesia with ketamine 10% (1 mL/kg) and xylazine 2% (0.1 mg/kg) (Agener União Saúde Animal, Brazil), and all efforts were made to minimise suffering. After coagulation, blood was centrifuged at 1,500 g for 10 min. Serum was removed and stored at −20°C.

Sera samples from the immunised mice were used in immunoassays (ELISA) for detection of specific IgG1, IgG2a and IgG3 antibodies. For this purpose, polystyrene 96-well plates (Greiner, USA) were coated with 100 μL of *C. pseudotuberculosis* T1 strain supernatant diluted 1:100 [15] (for G3 sera samples) or 0.125 ug/mL of rCP40 (for G2 sera samples) or a combination of both antigens (for G4 and G5 sera samples). All antigen dilutions were made in bicarbonate-carbonate buffer (pH 9.6). Plates were incubated at 4°C for 18 h. After this period, plates were washed twice with PBS-T (1X PBS, pH 7.4, 0.05% Tween 20) and blocked with 200 μL/well of 5% defatted milk in PBS-T for 2 h at 37°C. After this blocking step, the wells were washed five times with PBS-T and then 100 μL/well of sera samples from mice diluted 1:100 in PBS-T were added in duplicate. After one hour of incubation at 37°C and five washes with PBS-T, 50 μL/well of an anti-mouse antibody against IgG1, IgG2a or IgG3 (Invitrogen Life Technologies, USA) diluted 1:5000 was added. The plates were again incubated for 45 min at 37°C and then washed five times with PBS-T. After the washes, 50 μL/well of TMB developing solution (10 mL citric-phosphate buffer pH 5.1, 4 mg of orthophenylenediamine, 4 μL of H_2_O_2_) was added. The reaction was stopped by adding 25 μL of 4 N H_2_SO_4._ The absorbance was measured at 450 nm using an ELISA reader (Thermo Plate TP Reader, Thermo, USA).

### Statistical analysis

Statistical analyses were performed using GraphPad Prism version 6.0 for Windows (GraphPad Software, USA). The results of intracellular viability and specific immunoglobulins production were expressed as the means ± standard deviations. Differences between IgG production in the different groups were calculated by one-way ANOVA followed by the Tukey post-test test. The data were considered statistically significant when p < 0.05.

## Results

### Bacterial interaction assays with murine J774 cells

**Figure 1:**
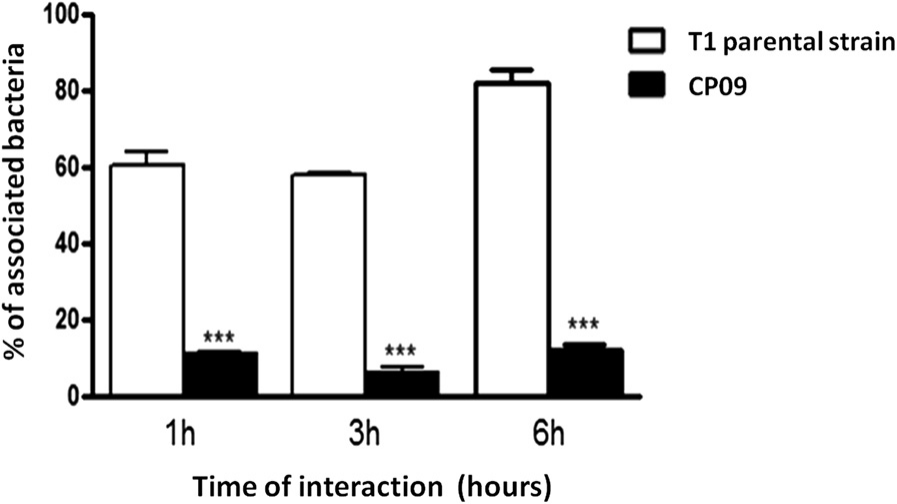
**Cellular invasion pattern of** ***C. Pseudotuberculosis*** **strain CP09 after 1, 3 and 6 hours of interaction with J774 cells.** The results are expressed as the means ± standard deviation of two representative experiments performed in quadruplicate. ***Significant reduction of cellular invasion versus T1 wild-type (wt) strain cellular invasion ; p < 0.5 (ANOVA, Tukey).

Analysing the results presented in Figure 1, it can be observed that the mutation harboured by the CP09 mutant had a major influence on the ability of this strain to adhere to J774 monolayers. After 6 hours of interaction, the rate of CP09 cell invasion in J774 cells was only 20% of the rate of cellular invasion achieved by the T1 parental strain at the same time point.

### Evaluation of the IgG levels

**Figure 2:**
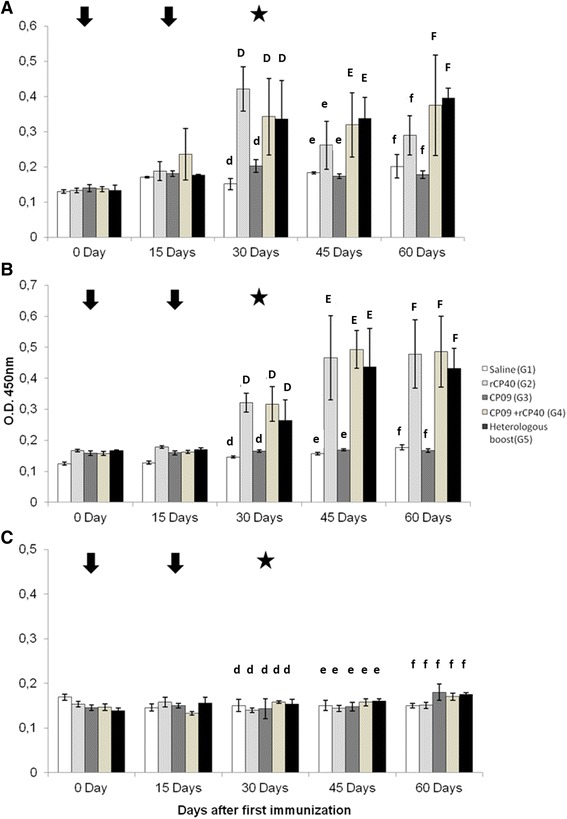
**Total specific IgG isotype levels in immunised mice with different vaccine formulations.** The sera of immunised mice were collected 0, 15, 30, 45, and 60 days post-immunisation and tested by ELISA for the presence of specific IgG1 **(A)**, IgG2a **(B)** and IgG3 **(C)**. The results are presented as the means and standard deviation (bars) for each experiment with ten animals per group. Significant differences between IgG production in the different groups was calculated employing one-way ANOVA followed by the Tukey post-test. D,d: groups significantly different on day 30 (p < 0.05); E,e: groups significantly different on day 45 (p < 0.05); F,f: groups significantly different on day 60 (p < 0.05). Arrows correspond to the two immunisations (days 0 and 15), and stars indicate the challenge with the virulent strain (Day 30). All bleedings were performed before immunisations and challenge.

Immunisations with the combination of rCP40 and CP09 induced the production of IgG1 in all immunised groups, as shown in Figure 2a. The results demonstrated that all groups on days 0 and 15 post-immunisation (p.i.) showed no significant IgG1 production (p > 0.05). On day 30, the G2, G4 and G5 groups showed significantly higher levels of IgG1 than groups G1 and G3 (p < 0.05). On days 45 and 60, after the challenge with the virulent strain, G4 and G5 groups showed significantly higher concentrations of IgG1 than the control and G3 groups (p < 0.05). No significant IgG1 production was observed in the G2 group after challenge.

Figure 2b shows the results obtained from the ELISA assay for the analysis of IgG2a production. On day 30 p.i., G2, G4 and G5 groups showed significantly higher concentrations of IgG2a (p <0.05) than groups G1 and G3. The same trend was observed in these groups after the MIC-6 strain challenge at 45 and 60 days p.i..

No significant IgG3 production was observed in any group, either before or after the virulent challenge (Figure 2c).

### Determination of protection levels

**Figure 3:**
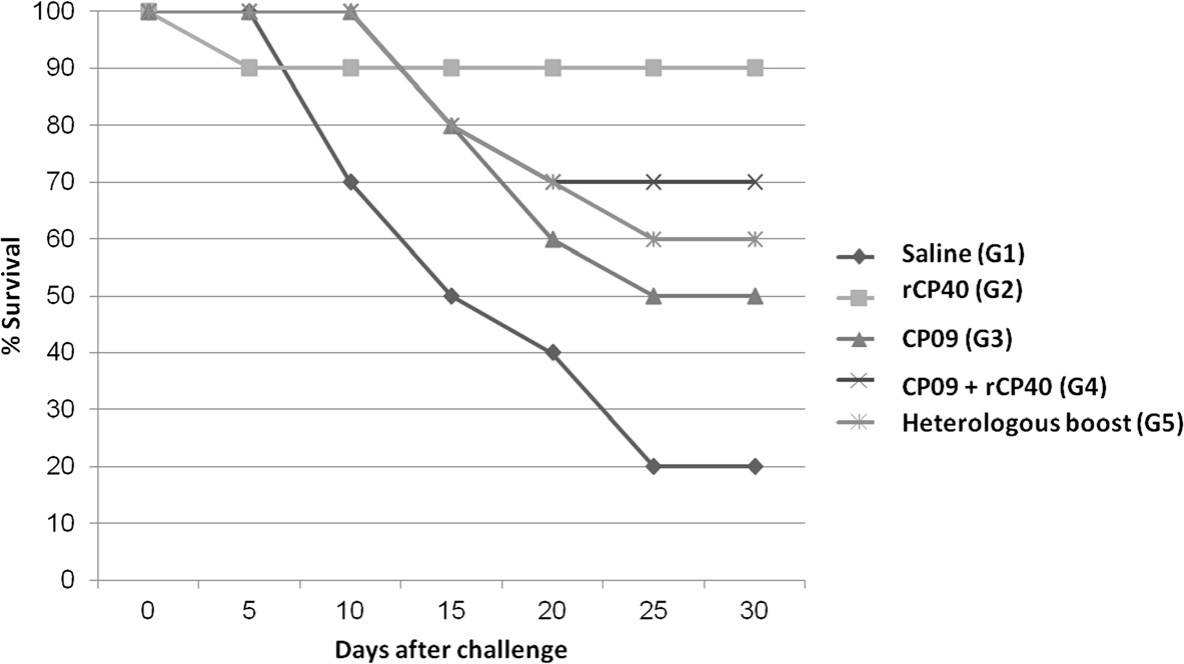
**Graphical representation of the survival of immunised mice after virulent challenge.** The data show the results of an experiment using 10 mice per group. Survival was monitored for 30 days after challenge.

The survival curve of mice immunised with vaccine formulations and challenged with *C. pseudotuberculosis* strains is presented in Figure 3. The animals were evaluated for thirty days after the challenge. In these trials, 10 mice in each group were monitored daily, and mortality events were recorded. Animals from the G2, G3, G4, and G5 groups showed, respectively, 90, 50, 70 and 60% survival 30 days after infection with the *C. pseudotuberculosis* MIC-6 wild strain.

## Discussion

Recombinant proteins and live attenuated vaccines are considered promising tools for the development of new vaccine models. In this regard, Dorella et al. [7], using a reporter transposon-based system, identified 34 out of 1,500 mutants that presented detectable alkaline phosphatase (PhoZ) activity. This feature was associated with 21 *C. pseudotuberculosis* loci that encode fimbrial and transport proteins. One of these mutants, CZ171049 (now called CP09) showed promising protective results in pilot trials and was suggested for further investigation in attenuation and immunisation-challenge trials.

In this study, we conducted a cellular adhesion experiment to observe how the mutation in the CP09 strain affected its capacity to adhere and infect murine macrophage cells. The results showed a significantly reduced capacity for cell adhesion and invasion, corroborating the genetic fimbrial mutant profile previously observed by Dorella et al. [7]. Clayton et al. [16] observed that mutations in *Salmonella enterica* fimbrial subunits genes led to reduced intestinal colonisation in chickens. Additionally, work by van der Velden et al. [17] showed that multiple fimbrial adhesions are required for a fully virulent profile in a murine model of *Salmonella typhimurium* infection. This attenuated cellular adhesion profile was confirmed for the *C. pseudotuberculosis* CP09 strain, and other experiments were conducted with the objective of determining the immunogenicity and protective potential of this mutant in a caseous lymphadenitis model in mice.

The development of cellular and humoral immune responses depends on the set of cytokines produced by various cells, including CD4^+^ and CD8^+^ T cells. When a naive T helper (Th) cell is activated by dendritic cells, it can differentiate into one of three distinct types of effector Th cells called T helper 1 (Th1), 2 (Th2) and 17 (Th17). Th1 cells are mainly involved in immunity against intracellular pathogens, and aid in the activation of macrophages and cytotoxic T cells and the production of opsonising and complement activator immunoglobulin isotypes, such as IgG2a in mice. Th2 cells are involved primarily in immunity against extracellular pathogens, such as multicellular parasites, and assist B cells in producing neutralising antibodies (IgG1 and IgG3) [18,19].

Based on the results presented herein, the G2 (rCP40), G4 (rCP40 + CP09) and G5 (prime-boost) groups had a very significant induction of IgG1, mainly from day 30 after the start of the trail. The G2 group did not presented presented significant IgG1 levels after the challenge (45 and 60 days after primary immunisation). These results suggest that the groups treated with inoculation solutions containing the mutant strain presented an immune response mediated by Th2 lymphocytes, as can be indicated by significant IgG1 production [20].

Although the protective role of IgG2a in this disease model is largely unknown, this IgG subtype is thought to modulate the immune response by inducing phagocytosis by neutrophils and macrophages and activating the complement system and cytotoxicity mediated by antibody-dependent cells. IgG2a production requires the induction of IFN-gamma by Th1 and NK cells [21-23]. Our results indicate that G2, G4 and G5 groups presented significant IgG2a production thirty days after the first immunisation, with a perceptible boost in its concentration after challenge with the recombinant strain; the group inoculated with the CP09 mutant strain alone was not able to produce specific IgG2a. Thus, significant IgG2a production in the above groups demonstrates the ability of the rCP40-saponin formulation to generate a Th1 adaptive immune response and memory. The Th1 immune response is responsible for the elimination of intracellular pathogens such as *C. pseudotuberculosis* [24], suggesting that the type of response induced in animals inoculated with the recombinant protein is consistent with the immune activity responsible for the control of intracellular bacteria.

According to Marini et al. [25], immune responses with high levels of IgG1, IgG2a and IgG3 in the serum are important mechanisms that help eliminate microorganisms. This indicates that our results are promising for the development of an effective vaccine against CLA. Vaccine formulations used in G2 (rCP40), G4 (rCP40 + CP09) and G5 (heterologous prime-boost) groups showed more significant results with respect to the level of specific immunoglobulins generated against rCP40, a recombinant protein that is considered to be an important *C. pseudotuberculosis* virulence factor in genomic and bioinformatics surveys [26].

A 90% protection rate was obtained in animals immunised with rCP40 alone, which is consistent with earlier work from Walker et al. [8]. These authors used the native semi-purified CP40 to evaluate antibody levels generated before and after infection in sheep and observed that this native 40 KDa protein provided an 82% protection rate, significantly reducing lung lesions. The protection in animals immunised only with CP09 mutant strain resulted in 50% survival after virulent challenge, which is in agreement with previously published results [7]. Ribeiro et al. [12] described a high protection rate (80%) in a murine model when using an iron acquisition-deficient mutant obtained with the same methodology employed to develop the CP09 mutant, showing that *C. pseudotuberculosis* live attenuated mutants can be promising tools for protection against caseous lymphadenitis.

Animals in groups G4 (CP09 + rCP40) and G5 (prime-boost) had survival rates of 70 and 60%, respectively. These two vaccine formulations had not been previously tested, so there is no work in the literature describing aspects of survival. After evaluating the results, it was observed that the survival rates of these groups were intermediate compared with those found in the G2 and G3 groups, which were vaccinated with only rCP40 (90% survival) and CP09 (50% survival), respectively.

The fact that CP40 recombinant protein alone led to a protection rate of 90% whereas the combination of rCP40 and CP09 mutant showed only a 70% protection can be explained by a differential complexation of the CP40 protein with the saponin adjuvant in the formulation using only the recombinant protein, when compared to the CP09 mutant-CP40 protein immunogen, which can lead to a different availability of the antigen to the immune system cells. A similar situation was seen by Keyburn et al. [27,28] when working with vaccines to *Clostridium perfringens*; they described that the protection rate achieved with the recombinant NetB protein alone was different from the one observed with the combination of the protein with the *C. perfringens* toxoid.

Taken together, these results suggest that vaccine formulations using the recombinant protein CP40 alone or in combination with the live recombinant strain CP09, or both antigens used in a prime-boost strategy, result in satisfactory survival percentages and antibody production. However, only the groups that had rCP40 in the inoculation solution presented an IgG2a response, and the group inoculated only with rCP40 was not able to induce an IgG-based humoral response, even after the challenge. These results indicate that rCP40 was able to induce a Th1 immune response, and the higher levels of protection in the G2 group can be observed as a result of this type of cellular immune response. All the groups stimulated with the combination of rCP40 with CP09 had a significant IgG1 response, indicating a Th2 response. The finding that the marked Th2 response elicited a low protection rate in those groups can be explained by the bacterial attenuation profile, which showed a reduced capacity of the mutant to adhere and invade murine cells. This reduced invasion can result in a defective presentation of bacterial antigens through a Major Histocompatibility Class (MHC) I pathway [21] because these antigens are not present in sufficient levels in the intracellular compartment. In this way, a Th2 response, represented by significant IgG1 production, prevailed, and the amount of Th1 cytokines was potentially insufficient to induce an adequate cellular response against the virulent *C. pseudotuberculosis* strain.

## Conclusions

The *C. pseudotuberculosis* CP09 live attenuated strain was not able to induce a humoral immune response when inoculated alone in mice; IgG production could only be observed when the mutant strain was combined in a vaccine formulation with the CP40 recombinant protein. The protection level was higher when the vaccine solutions included only the recombinant protein and was associated with a Th1 response in the absence of a Th2 profile after challenge, as seen by the IgG isotype production in immunised mice. Thus, rCP40 associated with a saponin adjuvant is a promising vaccine formulation in the control of caseous lymphadenitis.

## References

[1] Dorella FA, Pacheco LGC, Oliveira SC, Miyoshi A, Azevedo V (2006). *Corynebacterium pseudotuberculosis*: microbiology, biochemical properties, pathogenesis and molecular studies of virulence. Vet Res.

[2] Seyffert N, Guimarães AS, Pacheco LG, Portela RW, Bastos BL, Dorella FA, Heinemann MB, Lage AP, Gouveia AM, Meyer R, Miyoshi A, Azevedo V (2010). High seroprevalence of caseous lymphadenitis in Brazilian goat herds revealed by *Corynebacterium pseudotuberculosis* secreted protein-based ELISA. Res Vet Sci.

[3] Baird GJ, Fontaine MC (2007). *Corynebacterium pseudotuberculosis* and its role in ovine caseous lymphadenitis. J Comp Pathol.

[4] Radostits OM, Gay CC, Hinchcliff KW, Constable PD (2007). Veterinary Medicine: A textbook of the diseases of cattle, horses, sheep, pigs, and goats.

[5] Smith MC, Sherman DM (1994). Goat Medicine.

[6] Windsor PA (2011). Control of Caseous Lymphadenitis. Vet Clin Food Anim.

[7] Dorella FA, Estevam EM, Pacheco LGC, Guimarães CT, Lana UGP, Gomes EA, Barsante MM, Oliveira SC, Meyer R, Miyoshi A, Azevedo V (2006). *In vivo* insertional mutagenesis in *Corynebacterium pseudotuberculosis:* an efficient means to identify DNA sequences encoding exported proteins. Appl Environ Microbiol.

[8] Walker J, Jackson HJ, Eggleton DG, Meeusen E, Wilson MJ, Brandon MR (1994). Identification of a novel antigen from *Corynebacterium pseudotuberculosis* that protects sheep against caseous lymphadenitis. Infect Immun.

[9] Stanford K, Brodgen KA, McClelland LA, Kozub GC, Audibert F (1998). The incidence of caseous lymphadenitis in Alberta sheep and assessment of impact by vaccination with commercial and experimental vaccines. Canad J Vet Res.

[10] Chaplin PJ, De Rose R, Boyle JS, McWaters P, Kelly J, Tennent JM, Lew AM, Scheerlinck JPY (1999). Targeting improves the efficacy of a DNA vaccine against *Corynebacterium pseudotuberculosis* in sheep. Infect Immun.

[11] Moura-Costa LF, Bahia RC, Carminati R, Vale VLC, Paule BJA, Portela RW, Freire SM, Nascimento I, Schaer R, Barreto LMS, Meyer R (2008). Evaluation of the humoral and cellular immune response o different antigens of *Corynebacterium pseudotuberculosis* in Canindé goats and their potential protection against caseous lymphadenitis. Vet Immunol Immunopathol.

[12] Ribeiro D, Rocha FS, Leite KM, Soares SC, Silva A, Portela RW, Meyer R, Miyoshi A, Oliveira SC, Azevedo V, Dorella FA (2014). An iron-acquisition-deficient mutant of *Corynebacterium pseudotuberculosis* efficiently protects mice against challenge. Vet Res.

[13] Sambrook J, Russel DW (2001). Molecular Cloning – A Laboratory Manual.

[14] Ramos CRR, Abreu PAE, Nascimento ALTO, Ho PL (2004). A high-copy T7 *Escherichia coli* expression vector for the production of recombinant proteins with a minimal N-terminal His-tagged fusion peptide. Braz J Med Biol Res.

[15] Rebouças MF, Loureiro D, Bastos BL, Moura-Costa LF, Hanna AS, Azevedo V, Meyer R, Portela RW (2013). Development of an indirect ELISA to detect *Corynebacterium pseudotuberculosis* specific antibodies in sheep employing T1 strain culture supernatant as antigen. Braz J Vet Res.

[16] Clayton DJ, Bowen AJ, Hulme SD, Buckley AM, Deacon VL, Thomson NR, Barrow PA, Morgan E, Jones MA, Watson M, Stevens MP (2008). Analysis of the role of 13 major fimbrial subunits in colonisation of the chicken intestines by *Salmonella enterica* serovar Enteritidis reveals a role for a novel locus. BMC Microbiol.

[17] Van der Velden AW, Bäumler AJ, Tsolis RM, Heffron F (1998). Multiple fimbrial adhesins are required for full virulence of *Salmonella typhimurium* in mice. Infect Immun.

[18] Alberts B, Johson A, Lewis J, Raff M, Roberts K, Walter P (2007). Molecular Biology of the Cell.

[19] Dong C (2006). Diversification of T-helper-cell lineages: finding the family root of IL-17-producing cells. Nat Rev Immunol.

[20] Machado PRL, Araujo MIAS, Carvalho L, Carvalho EM (2004). Immune response mechanisms to infections. An Bras Dermatol.

[21] Abbas AK, Lichtman AH, Pillai S (2012). Cellular and Molecular Immunology.

[22] Adame-Gallegos JR, Shi J, McIntosh RS, Pleass RJ (2012). The generation and evaluation of two panels of epitope-matched mouse IgG1, IgG2a, IgG2b and IgG3 antibodies specific for *Plasmodium f*al*ciparum* and *Plasmodium yoelii* merozoite surface protein 1–19 (MSP119). Exp Parasitol.

[23] Lumsden JM, Nurmukhambetova S, Klein JH, Sattabongkot J, Bennett JW, Fox SRF, Reed SG, Ockenhouse CF, Howard RF, Polhemus ME, Yadava A (2012). Evaluation of immune responses to a *Plasmodium vivax* CSP-based recombinant protein vaccine candidate in combination with second-generation adjuvants in mice. Vaccine.

[24] Basso B (2013). Modulation of immune response in experimental Chagas disease. World J Exp Med.

[25] Marini V, Moretti E, Bermejo D, Basso B (2011). Vaccination with *Trypanosoma rangeli* modulates the profiles of immunoglobulins and IL-6 at local and systemic levels in the early phase of *Trypanosoma cruzi* experimental infection. Mem Inst Osw Cruz.

[26] Trost E, Ott L, Schneider J, Schröder J, Jaenicke S, Goesmann A, Husemann P, Stoye J, Dorella FA, Rocha FS, Soares C, D’Afonseca V, Miyoshi A, Ruiz J, Silva A, Azevedo V, Burkovski A, Guiso N, Join-Lambert OF, Kayal S, Tauch A (2010). The complete genome sequence of *Corynebacterium pseudotuberculosis* FRC41 isolated from a 12-year-old girl with necrotizing lymphadenitis reveals insights into gene-regulatory networks contributing to virulence. BMC Genomics.

[27] Keyburn AL, Portela RW, Sproat K, Ford ME, Bannam TL, Yan X, Rood JI, Moore RJ (2013). Vaccination with recombinant NetB toxin partially protects broiler chickens from necrotic enteritis. Vet Res.

[28] Keyburn AL, Portela RW, Ford ME, Bannam TL, Yan XX, Rood JI, Moore RJ (2013). Maternal immunization with vaccines containing recombinant NetB toxin partially protects progeny chickens from necrotic enteritis. Vet Res.

